# Remote sensing imagery detects hydromorphic soils hidden under agriculture system

**DOI:** 10.1038/s41598-023-36219-9

**Published:** 2023-07-05

**Authors:** Fellipe A. O. Mello, José A. M. Demattê, Henrique Bellinaso, Raul R. Poppiel, Rodnei Rizzo, Danilo C. de Mello, Nícolas Augusto Rosin, Jorge T. F. Rosas, Nélida E. Q. Silvero, Heidy S. Rodríguez-Albarracín

**Affiliations:** 1 Department of Soil Science, Luiz de Queiroz College of Agriculture, University of São Paulo, Pádua Dias Av., 11, Postal Box 09, Piracicaba, São Paulo 13416-900 Brazil; 2 Coordination of Integrate Technical Assistance of Secretariat of Agriculture and Supply-CATI/SAA, Piracicaba Regional, Campos Salles Street, 507, Piracicaba, São Paulo State 13400-200 Brazil; 3 Department of Soil Science, Federal University of Viçosa, Peter Henry Rolfs Av.-University Campus, Viçosa, Minas Gerais 36570-900 Brazil

**Keywords:** Environmental impact, Hydrology

## Abstract

The pressure for food production has expanded agriculture frontiers worldwide, posing a threat to water resources. For instance, placing crop systems over hydromorphic soils (HS), have a direct impact on groundwater and influence the recharge of riverine ecosystems. Environmental regulations improved over the past decades, but it is difficult to detect and protect these soils. To overcome this issue, we applied a temporal remote sensing strategy to generate a synthetic soil image (SYSI) associated with random forest (RF) to map HS in an 735,953.8 km^2^ area in Brazil. HS presented different spectral patterns from other soils, allowing the detection by satellite sensors. Slope and SYSI contributed the most for the prediction model using RF with cross validation (accuracy of 0.92). The assessments showed that 14.5% of the study area represented HS, mostly located inside agricultural areas. Soybean and pasture areas had up to 14.9% while sugar cane had just 3%. Here we present an advanced remote sensing technique that may improve the identification of HS under agriculture and assist public policies for their conservation.

## Introduction

Over the past decades multiple environmental challenges were addressed and worldwide initiatives have been established to promote sustainable development practices^1^. The world’s growing population and the increase demand for food and water, while minimizing the impact on climate raised awareness on the effort to achieve Food, Water and Energy Security, Climate Change Abatement, Biodiversity Protection and Ecosystem Service Delivery^2,3^. The soil has an important role on the achievement of such goals, but so far has been poorly applied in models to investigate these global challenges^4^. The lack of soil knowledge and the advance of its degradation caused by agriculture pose a global threat as the population is estimated to be 9 billion by the middle of the twenty-first century^5,6^.

Brazil has 41% of its area dedicated to agriculture (351 million ha)^7^, which increased 71 million ha in the last 33 years to cattle ranching and agriculture activities^8^. Naturally, fragile environments are pressured by this expansion, since agriculture activity releases chemicals considered toxic to flora, fauna, and human health^9,10^. In this regard, HS stand out as a fragile ecosystem responsible for hydrological and biogeochemical cycles with fauna and flora^11,12^. These soils are connected with the water table and represent a supplier for water recharge nutrients and sediments for riverine ecosystems^13^.

Saturated and waterlogged soils occupy around 6% of the Earth’s surface (2,1 million km^2^)^14,15^ and is the ecosystem with the highest damage rates^16^. Multiple studies presented ground water contamination through agricultural activities involving fertilizers, pesticides, and other chemical agents^17,18^, implying the fragility of such ecosystems and the impact of unregulated anthropic activities^19^. According to the Brazilian Forestry Code, the margin areas of a river, lake, and water source must be preserved with the natural vegetation^20^. However, the areas to be preserved start counting from the riverbed at the drought season, which end up excluding potential wetland soils that will only be affected during the flood season. These soils will naturally be included in agricultural sites at risk of getting contaminated, since they are difficult to map.

Identification and mapping of HS is vital to preserve these ecosystems from degradation regarding agriculture and other anthropic activity expansion. Multiple works combined digital geospatial information (i.e. satellite data) with machine learning algorithms to explore the relationship between soil and hydrology^21^. Some works focused on how the soil and landscape attributes affect channels morphometry^22–26^, while others explored the behavior within the soil^27–30^. Thompson 1997^31^ presented an early effort to quantitatively map HS through a soil color index using field sampling and terrain attributes derived from a digital elevation model. As remote sensing tools advanced, the mapping of wetlands^32–35^ and HS^36,37^ were possible due to the soil moisture effect on the reflectance intensity^38^.

To this end, agriculture expansion is bringing environmental pressure over HS and wetlands, demanding more detailed soil survey and mapping approaches. To our knowledge, there is no use of a remote sensing tool to assess HS located inside cultivated and not cultivated areas, enabling a better regulation of agriculture activity. Here we present a new remote sensing technique that allows the identification of HS at 30 m of spatial resolution for large areas. The method described as GEOS3^39^ uses a time-series of Landsat images to extract pixels with bare soil and aggregate them into a single synthetic soil image (SYSI). We used the Landsat 4 Thematic Mapper (TM) (1982–1993), Landsat 5 TM (1984–2012), Landsat 7 Enhanced Thematic Mapper Plus (ETM +) (1999–2018), and the Landsat 8 Operational Land Manager (OLI) (2013–2018). The technique combined more than 30 years of data in order to extract all the areas with bare soil, enabling a better regulation of agriculture activity and public policies towards environmental protection.

## Results and discussion

### Spectral characteristics of hydromorphic soils

**Figure 1:**
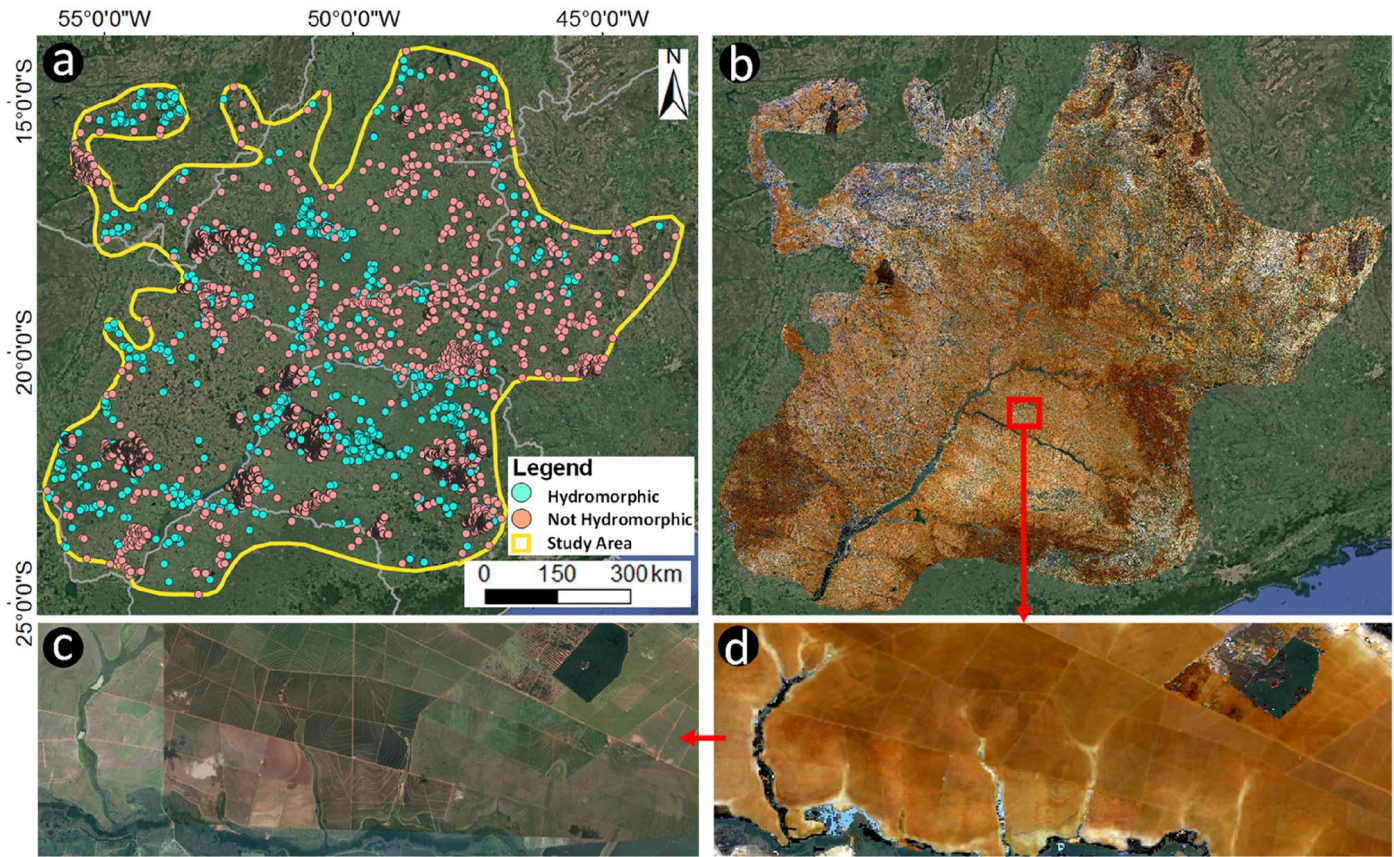
Expression of SYSI indicating hydromorphic soils across the study area, inside agricultural. (**a**) Study area with soil locations classified as hydromorphic and not hydromorphic. (**b**) Synthetic soil image (SYSI) for the study area. (**c**) Agricultural area covered with vegetation. (**d**) Uncovered hydromorphic features under the vegetation. Map created with ESRI ArcGIS 10.4.

We obtained 4954 georeferenced soil field samples of hydromorphic and not HS from the Brazilian Soil Spectral Library (BSSL)^40^ in an 863,577.9 km^2^ area in Brazil (Supplementary Fig. S1). This dataset contains samples with laboratory spectral analysis (350–2500 nm) and respective spectra acquired from bare soil satellite spectra from a synthetic soil image (SYSI)^39^. We also obtained 1579 additional synthetic observations of HS by combining field visits and qualitative analysis of SYSI, which served as reference for the digital sampling of locations with hydromorphic conditions (up to 40 cm depth). We selected field samples with laboratory spectra and classified as hydromorphic (Gleysols, Planosols, and Fluvisols) to evaluate their spectral behavior and to compare them with the SYSI spectra (Fig. 1a,b) and, to relate the SYSI spectra with agricultural areas covered with vegetation and uncovered hydromorphic features under the vegetation (Fig. 1b–d, respectively) (“Methods”).

**Figure 2:**
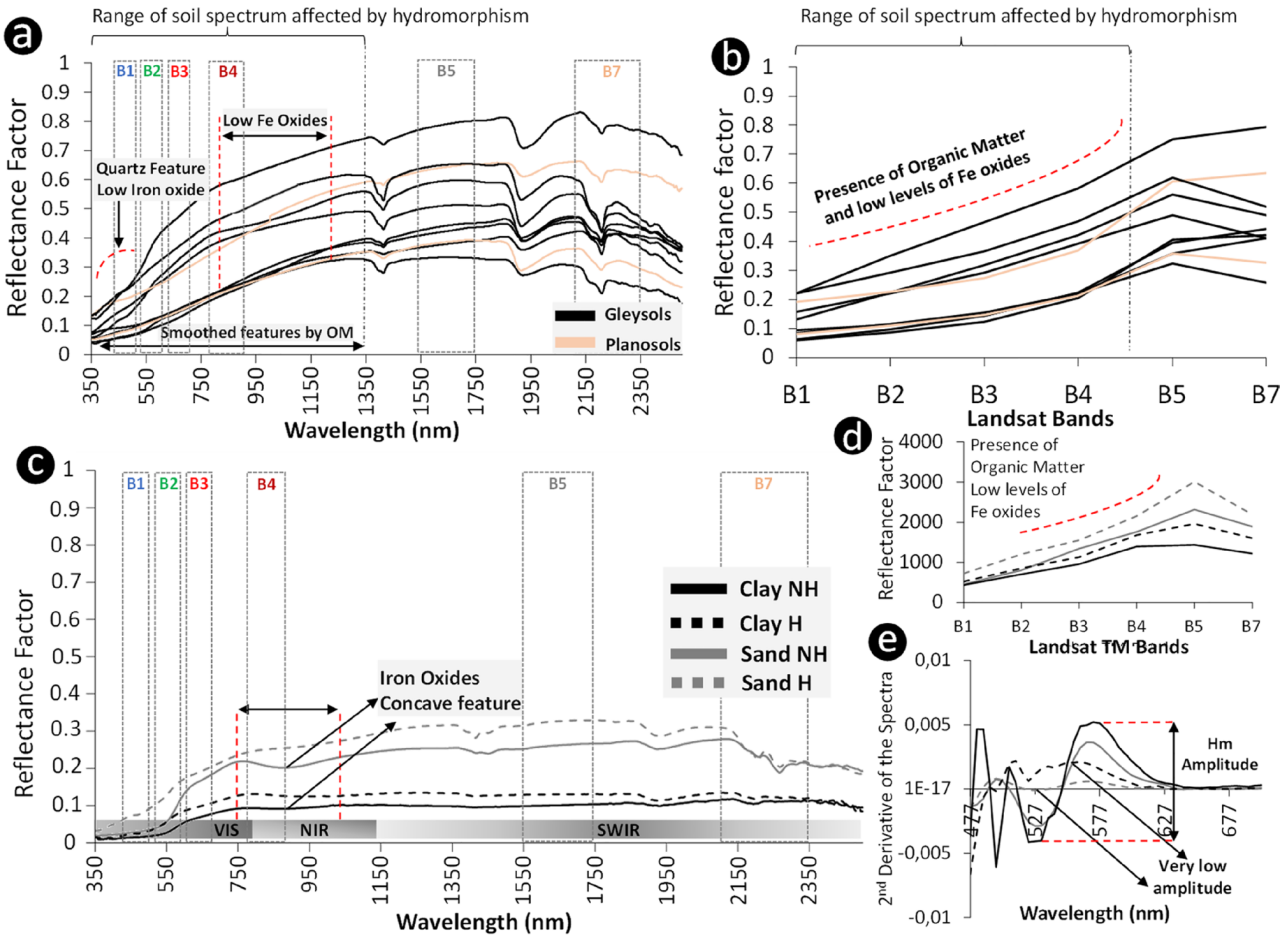
Laboratory and satellite topsoil spectra from hydromorphic and not hydromorphic soils. (**a**) Visible, near infrared, and short-wave infrared (350–2500 nm) laboratory topsoil spectra from A horizon of hydromorphic soils. (**b**) The spectral curves were convolved into the Landsat/TM bands to analyze the satellite spectral behavior. (**c**) Laboratory and satellite topsoil spectra from hydromorphic and not hydromorphic locations found in SYSI. (**d**) SYSI spectra collected at the same field locations from 1c. (**e**) Second derivative of the spectra to highlight the lower features of iron oxides at the hydromorphic locations.

The main characteristic observed in the laboratory spectra was the absence of a concave feature in the region of 900 nm, typical of the presence of iron oxyhydroxides^41^ (Fig. 1a). There is also an attenuation of features caused by the presence of organic matter in the soil between 350 and 1300 nm, with some of the curves showing a concave-rectilinear pattern between these bands (Fig. 2a). In addition, some samples presented a small convex feature between 350 and 450 nm. Therefore, the main spectral signature characteristics of HS were observed in the range from 350 to 1350 nm (Fig. 2a).

The spectral signatures presented in Fig. 1a were convolved for the Landsat bands in order to compare the spectra from the hydromorphic field samples with the SYSI spectral data (“Methods”). There was a concave-rectilinear pattern between Landsat band 1 to band 4 (Fig. 1b), indicating that part of the characteristics presented in the spectral signatures of laboratory data are reflected in the SYSI signatures, as, for instance, the attenuation caused by organic matter and absence of iron oxides, which are normal conditions for HS.

Four soil field samples were selected to illustrate the spectral differentiation between hydromorphic and not hydromorphic soils (Fig. 2c). Two samples were located in a toposequence with clayey soils and the other two sites were located in a toposequence with sandy soils (“Methods”). The HS had greater reflectance than not hydromorphic ones and did not present the typical concave shape around 900 nm regarding presence of Fe oxides (Fig. 2c). We applied the second derivative of the Kubelka–Munk function to the soil spectra^42^, which highlighted the absence of the typical hematite amplitude located between 520 and 580 nm (Fig. 2e). The result showed a slight peak around 560 nm, but not an absorption at 525 nm for HS samples and a higher reflectance between 350 and 450 nm for sandy soils (Fig. 2e).

When we observed the SYSI spectral signatures at the same four locations, we verified a higher reflectance for HS, and a slightly more concave shape between band 1 and band 4 (Fig. 2d). This was caused by a low presence of Fe oxides and higher contents of quartz (sand and silt fractions) in the soil surface, a result from permanent or periodic saturation of the soil by water^43^. The reflectance detected by the satellite only retrieves information about the surface, although it can be related to subsurface characteristics^44^. The surface of Gleysols (Hydromorphic) are not necessarily wet, contrary to the subsurface where water saturation promotes anoxic conditions. Nonetheless, the surface mineral and textural properties can be affected by the water saturation from below (groundwater) or from above (rain or irrigation water) and removal or reduction of Fe^3+^ to Fe^2+^^45^.

Based on the absence of Fe oxides features in the laboratory and SYSI spectra at the hydromorphic sites, we identified specific areas as hydromorphic in the synthetic image, which enabled its use for the classification of hydromorphic areas. The hydromorphic sites were often near drainage network systems, which are zones normally affected by groundwater level fluctuations and that concentrate the drained water from upper positions of the watershed (Supplementary Fig. S3). This observation suggested the influence of soil moisture in the reflectance response, causing differences in color intensity similar to the features found by^46^.

### Spatial prediction of hydromorphic soils

We combined the soil dataset with SYSI and a set of terrain attributes calculated with the Terrain Analysis in Google Earth Engine package^47^ (Supplementary Table S1). The resulting dataset was used to fit a random forest (RF) model in order to classify the areas as hydromorphic across the study area (Methods). The optimal model used RF and cross-validation as the resampling method, reaching an overall accuracy of 0.92 and Kappa coefficient of 0.77 for the binary classification of pixels as hydromorphic and not hydromorphic. We also evaluated the model with a confusion matrix, reaching a producer accuracy of 87% for the hydromorphic and 94% for the not hydromorphic class (Supplementary Table S2).

The model was able to correctly classify 6086 out of 6533 soil observations displaced across the study area. The boxplot analysis indicated a significant difference in slope, elevation, and horizontal curvature regarding hydromorphic and not hydromorphic classes (Supplementary Fig. S2a), which also manifested in the prediction performance with 98, 38, and 29% of importance for the model prediction (Supplementary Fig. S2b). SySI had a similar prediction importance for bands 2, 3, 4, 6, and 7, while the boxplot analysis showed significant differences for all satellite bands between hydromorphic and not hydromorphic soils (Supplementary Fig. S2a). SYSI band 1 (Blue) had the second highest contribution for the model with 69%, highlighting the influence of soil moisture in the reflectance response.

### Hydromorphic soil distribution

**Figure 3:**
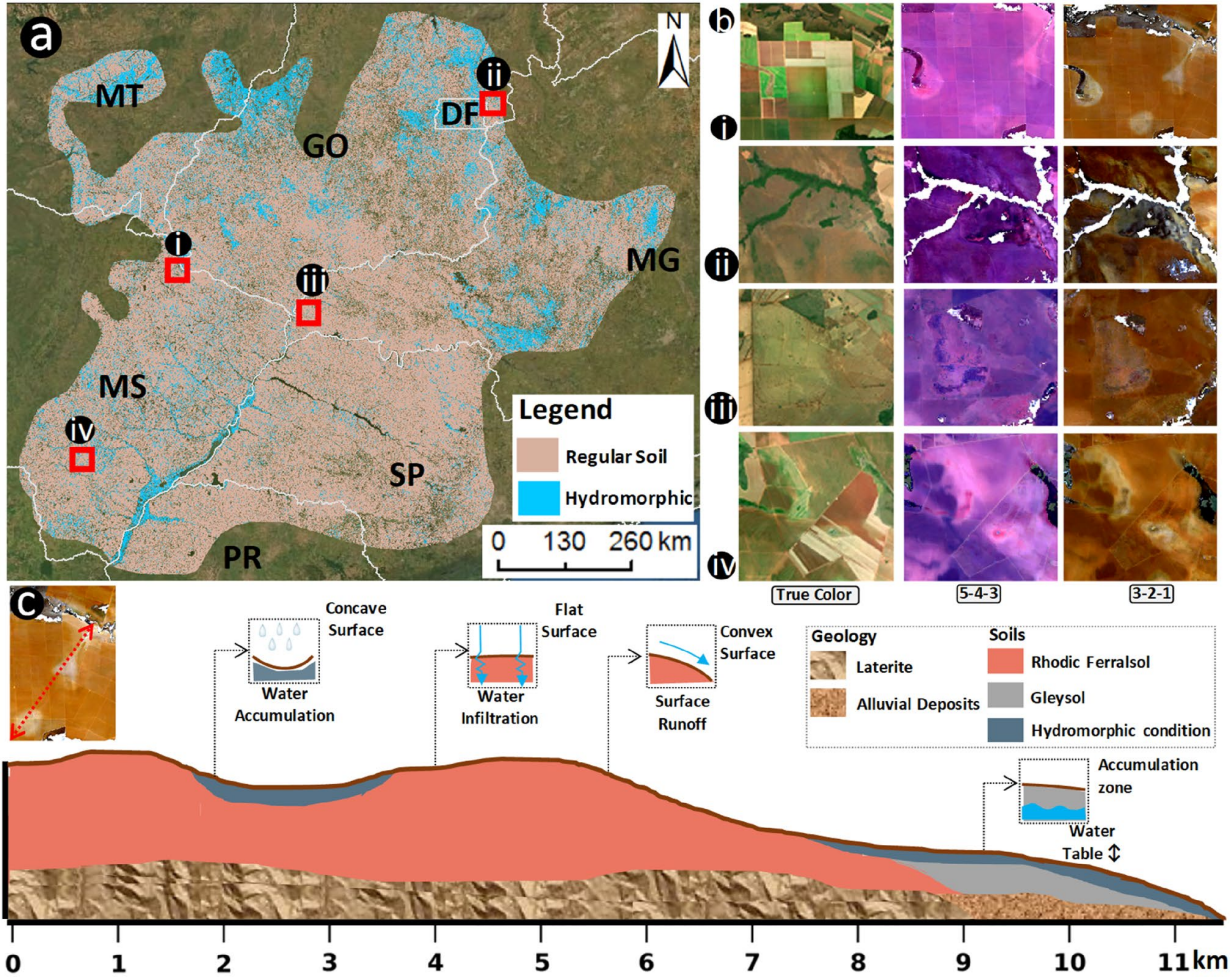
Results of the modelling and the relief pattern of areas classified as hydromorphic. (**a**) Predicted map of hydromorphic soils for the study area. (**b**) Indication of SYSI as a tool to identify hidden hydromorphic soils. (**c**) Toposequence extracted from area (i) indicating the relief positions of hydromorphic soils, the geology and soil classes. Map created with ESRI ArcGIS 10.4.

We selected four locations from the predicted map (Fig. 3a) and displayed them in true color composition, RGB 543 and 321 to observe and analyze the areas classified as hydromorphic (Fig. 2b). It was possible to identify some bright and darker features in the images, showing differences in the reflectance factor and the occurrence of HS at different landscape positions (Fig. 2b).

The differences in the reflectance factor usually indicates textural variation, Fe oxides presence, higher organic carbon content, soil moisture, and others^48^. Figure 2bi presented three possible HS, one surrounding a channel’s water source enhancing the possible area of Gleysols. In the same location there is an intermittent channel of second order, which is an overland flow path. Finally, a closed depression with concave landform that functions as an accumulation zone (Fig. 3bi). Figure 3bii represents a footslope, a flat landform next to a thalweg and normally influenced by groundwater level fluctuations. The Landsat 321 RGB composite showed an area with lower reflectance intensity at the footslope, indicating hydromorphism (Fig. 3bii). A flat area at the summit and an overland flow path with the same features of lower reflectance intensity were observed in Fig. 3biii, iv. These areas are displaced across the landscape, retaining water within the soil due to landforms or a soil characteristic that hampers water infiltration.

We plotted the relief profile in a toposequence to evaluate how the relief contributes for the formation of a HS (Fig. 3c). The HS occurred over two soil types, a Ferralsol and a Gleysol (Fig. 3c). The Ferralsols are weathered soils normally located at flat surfaces, which favor water infiltration and prevent the formation of drainage channels^45^. The Ferralsol was also located at the hillslope with convex surface, favoring surface runoff and drainage channel formation (Fig. 3c). The Gleysols are normally located at lower relief positions that constantly receives and accumulates sediment and water, favoring redoximorphic activity in the soils^49^.

### Land use over hydromorphic soils

**Figure 4:**
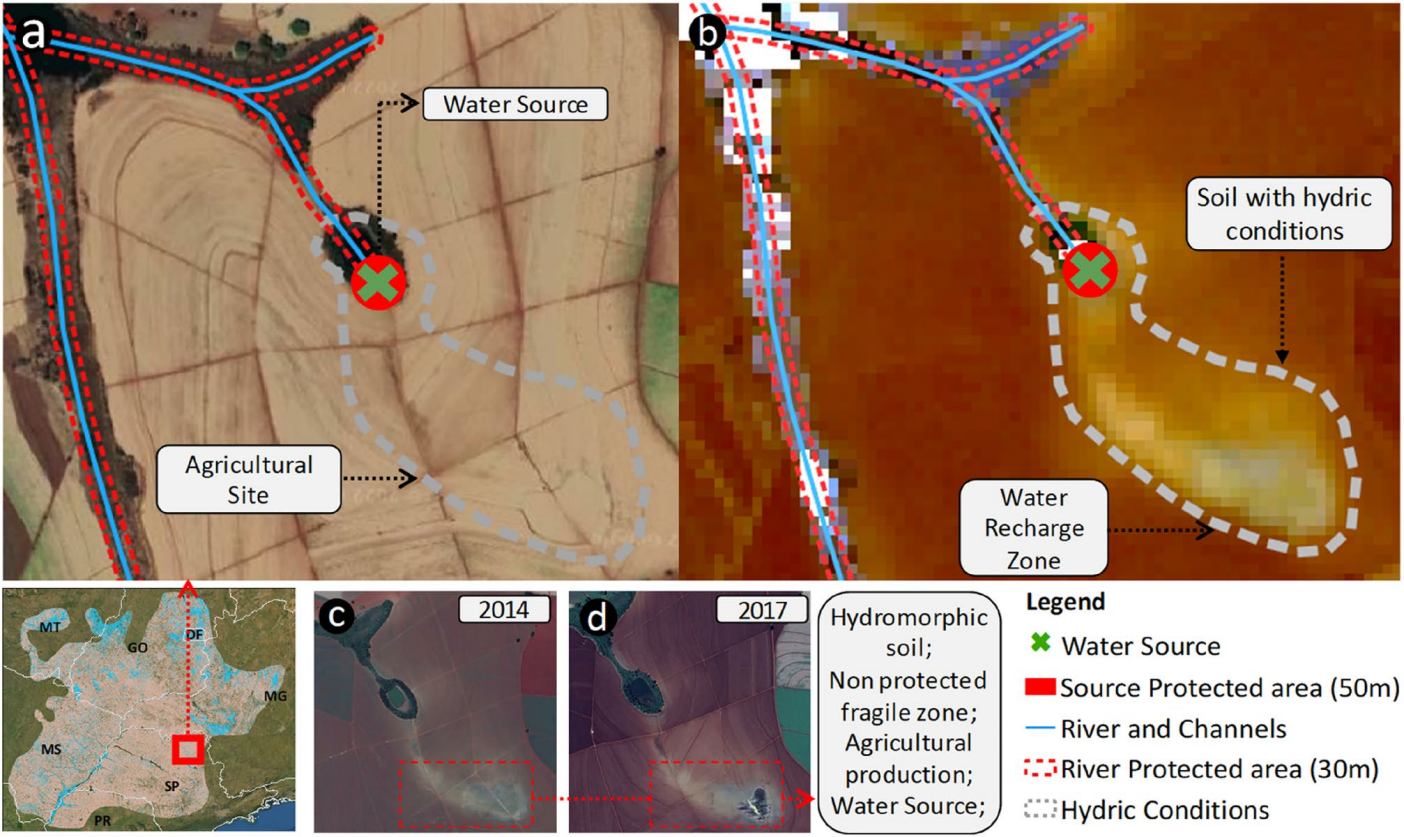
Example of hydromorphic soils within an agricultural field. (**a**) The supposed water source is protected with vegetation. (**b**) SYSI identifies abrupt difference in reflectance indicating hydromorphic conditions. (**c**,**d**) Landsat true color in 2014 and 2017 with exposed soil showing accumulation of water in the actual water source. Map created with ESRI ArcGIS 10.4.

Figure 4a shows an agricultural site with a Permanent Protected Area (PPA) surrounding the channels and the water source. However, a prolonged area with HS is under agriculture exploration (Fig. 4b). The HS is hidden under an agricultural area in contact with chemical fertilizers and pesticides, common for management practices. Agricultural practices over these soils can accelerate nutrient loss, affect particle aggregation, distribution and mineralogy of Fe oxides between particle-size fractions, and the interaction with organic matter stabilization^50,51^. Also, these soils are more fragile to receive pollution from herbicides and pesticides, reaching ground water. Two Landsat images from the same area in 2014 and 2017 indicate a different location for the water source and an intermittent channel connecting with the actual PPA (Fig. 4c,d). The technique identified these soils and showed that multiple hydromorphic areas are located at agricultural fields, promoting degradation (Table 1).

**Table 1:** Predicted areas of hydromorphic soils for the study area analyzed by federal states and land use classifications according to Souza et al. (2020)^8^.

Study area	TA (km^2^)	Area (km^2^)		
		*NH*	*H*	*H (%)*
	735,953,8	629,605,7	106,348,2	14.5

States	PA (%)	Area (km^2^)		
		*NH*	*H*	*H (%)*
Federal district	89.4	3074.2	2077.5	40.3
Goiás	57.9	162,072.2	35,116.5	17.8
Minas Gerais	22.8	112,925.2	20,646.0	15.5
Mato Grosso do Sul	44.8	137,812.8	22,293.0	13.9
Mato Grosso	5.8	37,488.6	14,617.5	28.1
Paraná	19.9	37,065.8	2646.1	6.7
São Paulo	59.7	139,166.9	8951.4	6.0

Land use	TA (km^2^)	Area (km^2^)		
		*NH*	*H*	*H (%)*
Sugar cane	86,872.4	84,209.1	2663.3	3.1
Soybean	113,553.9	97,037.2	16,516.7	14.5
Forest Plantation	19,771.9	17,728.0	2043.8	10.3
Pasture	267,460.2	227,509.8	39,950.3	14.9
Temporary Crop	11,439.8	10,015.0	1424.8	12.5
Perennial Corp	5345.4	4926.1	419.3	7.8

*TA* total area, *NH* not hydromorphic, *H* hydromorphic, *PA* percentage of area.

We found differences in the hydromorphic areas between federal states and land uses, requiring further investigations for the correct application Brazilian Forestry Code. Only 6% of the studied area belonging to the state of São Paulo were classified as hydromorphic, while Mato Grosso do Sul, Minas Gerais, and Goiás had up to 17.8% (Table 1). Mato Grosso had only 5.8% of its area included in this work, but had 28% classified as hydromorphic. In the world about 26% of the gleysols are agricultural lands^52^. The state of São Paulo is considered the Brazilian state with the greatest legal framework for environmental protection^53,54^. In 1994, it was the first state to regulate the use of floodplain areas. In addition, it has a law that regulates the use and conservation of agricultural soil (“Soil law”) since 1988. In addition to São Paulo, only the states of Paraná (1984), Espírito Santo (2001) and Rio Grande do Sul (2015), have similar regulation^55^.

The sugar cane areas had 3.1% of hydromorphic areas, an indication of adequate ap-plication of PPA for a major agricultural activity in the country^56^. On the other hand, pastures across the study area had 14.9% of hydromorphic areas, enhancing the risks of contamination since this land use is normally degraded due to due to low grass productivity and inadequate grazing management^56^. The soybean areas also had around 14% of areas classified as hydromorphic, followed by forest plantation and temporary crops with 10 and 12%, respectively (Table 1). Soybean is the predominant crop system in the states of Mato Grosso, Mato Grosso do Sul, and Goiás^57^, which were the states with higher percentage of HS (Table 1). Sugar cane is the predominant agricultural system in São Paulo state, which explains the lower occurrence of HS in this state.

The observed differences in HS regarding federal states and land use suggest a further investigation on the application of the Brazilian Forest Code. The modification of hydromorphic environments increases the emission of gases and changes the soil dynamics^58^, being important areas for springs and water bodies^59^, suggesting stronger preservation of these areas. Multiple initiatives discussed the real benefits and limitations of the defined areas of preservation, discussing whether they should be larger in order to preserve natural resources. However, this technique was able to identify multiple channel networks (intermittent and perennial) and water sources inside agricultural sites (Supplementary Fig. S3). In the current Forest Code, the delimitation of PPAs occurs from the regular riverbank, unlike the previous code (Forest Code of 1965), in which the largest riverbank was considered^60^. This change may contribute to an increase in the agricultural use of HS areas, especially in floodplain areas. Finally, more remote sensing tools must be applied to monitor the control of natural resources, which should be preserved and well managed in order to avoid future degradation.

## Methods

### Soil inventory

We obtained 4715 georeferenced soil field samples of hydromorphic and not hydromorphic soils from the Brazilian Soil Spectral Library (BSSL) in an 735,953.8 km^2^ area across the Southeast and Midwest regions of Brazil (Supplementary Fig. S1). The study area comprises tropical and subtropical climates classified as Savanna (Aw), Subtropical highlands (Cwb), and Humid subtropical (Cfa, and Cwa) according to the Köppen climate classification. The rainfall varies between 1000 to 2200 mm per year and the mean annual temperature between 18 and 24 °C^61^.

Most of these soil samples were acquired from traditional soil surveys, which consist of a soil specialist using conventional soil surveying methods to select locations based on pedogeomorphological relationships for profile description and sampling^62^. We excluded the Gleysols and Planosols observations from the dataset to keep only not hydromorphic soils. We also obtained 1341 additional synthetic observations of HS by combining field visits and visual analysis of a synthetic soil image (SYSI)^39^, which served as reference for the digital sampling of locations with hydromorphic conditions (up to 40 cm depth). These synthetic samples were based on geographical location and SYSI’s reflectance intensity (indicating soil moisture). Finally, we reached a dataset with two categories, as follows: (i) not hydromorphic soils, composed by Acrisols, Cambisols, Chernozems, Podzols, Ferralsols, Luvisols, Leptosols, Arenosols, Regosols, and Nitisols; (ii) HS, composed by synthetic samples (field and digital).

### Remote and proximal spectroscopy analysis

We used an external soil dataset with proximal spectral information (350–2500 nm) of Gleysols and Planosols (Hydromorphic) from three works^40,63,64^. We used these data to identify the features related to hydromorphism, such as organic matter presence, absence of Fe oxide features, and the spectra intensity. We analyzed 10 samples collected at 0–20 cm depth to compare the laboratory and satellite spectra. The method for the spectroscopic analysis is described by Demattê et al.^65^. Thus, we convolved the soil spectra for the Landsat bands^42^, in order to compare the spectra from the hydromorphic field samples with the SySI spectral signature.

After establishing the spectral signatures of HS, we selected two samples at locations where SySI had the hydromorphic conditions. These samples contained SYSI and laboratory spectral analysis (350–2500 nm) and were used to define if the changes in spectral intensity was in fact related to hydromorphism. We also applied the second derivative of the Kubelka–Munk function, as a way to highlight the changes in the spectra^42^. After confirming the spectral pattern (350–2500 nm) for HS, we selected 1341 locations with the same spectral features. SYSI showed a change in hue and intensity of colors at the locations with possible hydromorphic conditions. This pattern was normally observed next to drainage channels, at flat surfaces, and close to the water sources.

### Modelling

Soil formation is a result from the interaction of multiple factors such as climate, organisms (biota), relief (landscape processes), parent material (geology), and time^66^. Thus, we combined the soil dataset with a set of environmental variables related to soil forming factors (Supplementary Table S1) to fit a RF model.

We used two categories of environmental variables, the first is related to bare soil reflectance from satellite images and the second is related to the terrain. We implemented the Geospatial Soil Sensing System (GEOS3)^39^ to a time-series of Landsat images using the Google Earth Engine (GEE) platform^67^ in order to generate a 30 m spatial resolution Synthetic Soil Image (SYSI). SYSI has six spectral bands from blue to short-wave infrared regions at 30 m resolution (Supplementary Table S1).

The terrain attributes were calculated using the Terrain Analysis in GEE (TAGEE) package^47^. The package calculates multiple topographic variables from a Shuttle Radar Topography Mission (SRTM) digital elevation model (DEM) with a spatial resolution of 30 × 30 m. Finally, we overlapped the soil dataset with the TAGEE and SYSI attributes and sampled the matching locations to perform a boxplot analysis. The sampled data were plotted by class (hydromorphic and not hydromorphic) in order to analyze the distribution of values. The graphic was performed using the “ggplot” package in R software.

The RF algorithm was selected to perform the hydromorphic areas mapping, since its relevance for digital soil mapping^68,69^. RF estimates a user-specified number of decision trees by randomly sampling an existing dataset^70^. However, at each node construction, a random sample of the dependent variables is used. The resulting decision tree is used to estimate the out-of-bag error rate by predicting the value of the remaining unsampled data and comparing with the known results.

We performed a grid search to select the optimal hyperparameters, which were the maximum depth (150), maximum features (3), minimum samples leaf (1), minimum samples split (10), and number of trees (300). These parameters regulate the number of variables that can be randomly sampled in each split of the trees, the tree depth by setting the minimal number of samples for the terminal nodes, and the number of trees.

In order to calibrate the RF model, we tested three resampling methods coupled with the RF model using the caret package in R software. The first test used k-fold cross-validation (CV) method to fit the prediction models. CV is a resampling method used to fix optimistic results of the predictive effectiveness of regression equations. The method randomly divided the data in k groups, using k − 1 groups to fit a model, and one for validation^71,72^. The procedure is repeated k times, always leaving one group out of the calibration dataset. Afterwards, the results are summarized with the mean of the model scores. The second method was the bootstrapping, which is a data resampling technique for estimating the statistical parameters of an unknown distribution and a robust method for optimal model selection^73^. We also tested the out-of-bag resampling method which is a method of measuring the prediction error of random forests^74^.

The prediction performance of the data was accessed using the three default parameters of caret for classification models, being the number of randomly selected predictors (mtry), accuracy, and kappa coefficient. The mtry regulates the number of variables that can be randomly sampled in each split of the trees, which resulted in 2, 20, and 39. We used 300 trees for stable variable estimates.

As many environmental information were used as covariables to fit the RF model, we analyzed the variables’ importance through the mean decrease Gini index. The analysis helped indicating how was the contribution of the terrain, climate, and remote sensing variables.

### Spatial prediction and model validation

After testing the resampling methods and fitting the RF model, we selected the optimal model and used its parameters to predict the hydromorphic and not hydromorphic classes across the study area with the caret package in R software 3.4.0 (https://cran.r-project.org/bin/windows/base/)^75,76^. The resulting binary map was generated using the caret and raster packages in R software, and exported to ArcGIS 10.4. For further analysis. We calculated a confusion matrix for the optimal model and analyzed the errors of inclusion or commission errors (CE), errors of exclusion or omission errors (OE), user accuracy (UA), producer accuracy (PA), and global accuracy (GA)^77^.

We also evaluated the areas mapped as hydromorphic according to their relief position (infiltration or surface runoff environment), proximity to the channel network or water source, and according to the pattern registered by SYSI. We selected an area to analyze the distribution of HS across a toposequence, based on the soil-landscape relationship rules and the channel network patterns.

### Hydromorphic soils distribution over different land use/cover and federal states

With the digital map of HS, we were able to analyze the current land use situation of these soils. First, we masked the pixels classified as hydromorphic and exported them to a new raster in ESRI ArcGIS 10.4. The new raster was plotted over a land use and landcover map from the MapBiomas project. MapBiomas is a governmental initiative aimed to reconstruct annual land use and land cover information between 1985 and 2017 for Brazil based on random forest applied to Landsat archive using Google Earth Engine^8^. The dataset is available for download at their repository in GEE and at their website (https://mapbiomas.org/)^8^.

Finally, we quantified the areas of HS and identified the land uses at the areas. We also computed the total areas of HS for each federal state included in the study area. The result was presented in a table with the total area of HS for each land use class and federal state.

## Supplementary Information

Supplementary Figures.

Supplementary Tables.

## Data Availability

The hydromorphic soils map generated during the current study is available in raster format in the link: https://esalqgeocis.wixsite.com/english/felipe-mello-hydromorphic-soils.

## Code availability

The code to generate the final product of the hydromorphic soils map is available from the corresponding author upon reasonable request for the following reasons: (a) the script/code creates a proxy from which our research group is working on the development of new products that will be published in the near future.
