# Supplementary material for: Remote sensing imagery detects hydromorphic soils hidden under agriculture system
Source: Sci Rep. 2023 Jul 5;13:10897. doi: 10.1038/s41598-023-36219-9 (PMC10322844; doi:10.1038/s41598-023-36219-9)
Supplement: Supplementary file 1 — Supplementary Figures. [file 41598_2023_36219_MOESM1_ESM.docx]

Supplementary Figure 1| Study area located between the southeast and mid-west regions of Brazil. The map shows the point locations where soils were identified as hydromorphic and not hydromorphic, depending on their conditions analyzed in the field and through spectral analysis.

Supplementary Figure 2 | Boxplot analysis and importance of each environmental variable for the prediction performance. **a**, TAGEE and SySI values distribution according to the soil classification as H: hydromorphic and NH: not hydromorphic. DEM: digital elevation model; SLP: slope; NRT: northernness; EST: easternness; HCV: horizontal curvature; VCV: vertical curvature; SID: shape index; B1 to B7 refers to the Landsat bands used in SySI. **b**, Graphic of Variables importance for the prediction of hydromorphic soils with random forest (RF) model. The variables are separated by relief and satellite classes.

Supplementary Figure 3 | Observation of multiple hydromorphic soils inside agricultural sites with using SySI image with RGB composition 5-4-3. Map created with ESRI ArcGIS 10.4.
