# Supplementary material for: Remote sensing imagery detects hydromorphic soils hidden under agriculture system
Source: Sci Rep. 2023 Jul 5;13:10897. doi: 10.1038/s41598-023-36219-9 (PMC10322844; doi:10.1038/s41598-023-36219-9)
Supplement: Supplementary file 2 — Supplementary Tables. [file 41598_2023_36219_MOESM2_ESM.docx]

| Class | Attribute | Description | Unit | Reference |
| --- | --- | --- | --- | --- |
| Terrain | DEM | Elevation | meter | TAGEE^46^ |
|  | SLO | Slope | degree |  |
|  | NRT | Northernness | ND |  |
|  | EST | Easternness | ND |  |
|  | HCV | Horizontal Curvature | meter |  |
|  | VCV | Vertical Curvature | meter |  |
|  | SID | Shape Index | ND |  |
| Remote Sensing | B1 | Landsat Band 1 – Blue | Ref. factor | GEOS3^39^ |
|  | B2 | Landsat Band 2 – Green | Ref. factor |  |
|  | B3 | Landsat Band 3 – Red | Ref. factor |  |
|  | B4 | Landsat Band 4 – Near Infrared | Ref. factor |  |
|  | B5 | Landsat Band 5 – SWIR1 | Ref. factor |  |
|  | B7 | Landsat Band 7 – SWIR2 | Ref. factor |  |

Supplementary Table 1: Environmental variables used as predictors for the digital soil mapping (DSM).

ND: dimensionless.

Supplementary Table 2: Confusion matrix regarding the predicted classes of hydromorphic soils for the study area.

|  | Class | Reference | | Total | UA | OE | CE |
| --- | --- | --- | --- | --- | --- | --- | --- |
|  |  | H | NH |  |  |  |  |
| Predicted | H | **1027** | 299 | 1326 | 77% | 23% | 13% |
|  | NH | 148 | **5059** | 5207 | 97% | 3% | 6% |
|  | Total | 1175 | 5358 | 6533 |  |  |  |
|  | PA | 87% | 94% |  | **6086** |  |  |

UA: user accuracy; OE omission error; CE: commission error; PA: producer accuracy. Bold represents the sum of the major diagonal (The total correctly classified soil classes).
